# A Novel PEGylated Liposome-Encapsulated SANT75 Suppresses Tumor Growth through Inhibiting Hedgehog Signaling Pathway

**DOI:** 10.1371/journal.pone.0060266

**Published:** 2013-04-01

**Authors:** Yike Yuan, Yuwei Zhao, Shengchang Xin, Ni Wu, Jiaolin Wen, Song Li, Lijuan Chen, Yuquan Wei, Hanshuo Yang, Shuo Lin

**Affiliations:** 1 State Key Laboratory of Biotherapy and Cancer Center, West China Hospital, West China Medical School, Sichuan University, Chengdu, Sichuan, People's Republic of China; 2 Laboratory of Chemical Genomics, Shenzhen Graduate School of Peking University, Shenzhen, People's Republic of China; 3 Department of Molecular, Cell, and Developmental Biology, University of California, Los Angeles, Los Angeles, California, United States of America; King Faisal Specialist Hospital & Research Center, Saudi Arabia

## Abstract

The Hedgehog (Hh) pathway inhibitors have shown great promise in cancer therapeutics. SANT75, a novel compound we previously designed to specially inhibit the Smoothened (SMO) protein in the Hh pathway, has greater inhibitory potency than many of commonly used Hh inhibitors. However, preclinical studies of SANT75 revealed water insolubility and acute toxicity. To overcome these limitations, we developed a liposomal formulation of SANT75 and investigated its antitumor efficacy *in vitro* and *in vivo*. We encapsulated SANT75 into PEGylated liposome and the mean particle size distribution and zeta-potential (ZP) of liposomes were optimized. Using the Shh-light2 cell and Gli-GFP or Flk-GFP transgenic reporter zebrafish, we confirmed that liposome-encapsulated SANT75 inhibited Hh activity with similar potency as the original SANT75. SANT75 encapsulated into liposome exerted strong tumor growth-inhibiting effects *in vitro* and *in vivo*. In addition, the liposomal SANT75 therapy efficiently improved the survival time of tumor-bearing mice without obvious systemic toxicity. The pathological morphology and immunohistochemistry staining revealed that liposomal SANT75 induced tumor cell apoptosis, inhibited tumor angiogenesis as assessed by CD31 and down-regulated the expression of Hh target protein Gli-1 in tumor tissues. Our findings suggest that liposomal formulated SANT75 has improved solubility and bioavailability and should be further developed as a drug candidate for treating tumors with abnormally high Hh activity.

## Introduction

The hedgehog (Hh) signaling pathway is a major regulator for embryonic development and adult tissue homeostasis [1], [2]. When Hh ligands bind to Patched1 (Ptch1), the repressed co-receptor Smoothened (SMO) becomes active, allowing signal transmission resulting in upregulation of target genes such as Ptch1 and Gli1, which results in promoting cell proliferation and differentiation [3]–[5]. Aberrant Hh pathway has been associated with a broad spectrum of tumors such as lung, breast, prostate, ovarian and colorectal cancers [6]–[11]. In addition to cancers harboring Hh pathway-activating mutations that are Hh ligand independent, normal Hh ligand-dependent Hh activity have been proposed to stimulate tumor growth. In this case, cancer cells respond to Hh in an autocrine or juxtacrine manner. Moreover, the Hh secreted by ligand-dependent cancers is received in a paracrine manner by the surrounding stroma or cancer stem cells, which further feed back signals such as IGF, Wnt and VEGF to the tumor tissue to support its growth or survival [12],[13]. Therefore, inhibitors of the Hh pathway, including natural compound cyclopamine and other chemically optimized Hh antagonist compounds have great promise in anticancer therapy [13]. It's reported that blocking the Hh pathway by cyclopamine decreased the activation of oncogenic PI3K/Akt, NF-kB and MAPK pathways, inhibited tumor growth, angiogenesis, and suppressed pancreatic cancer invasion and metastasis through inhibiting EMT in the pancreatic cancer cells [14]–[16]. Recently, FDA has approved the first anti Hh drug for treating basal cell carcinoma [17]. Additional drugs based on cyclopamine are currently in Phase I and Phase II clinical trials [18].

SANT75 is a Hh antagonist that we designed to specially inhibit SMO protein through inducing conformational change of SMO [19], which exhibits stronger inhibiting effects in mammalian cells with the IC_50_ of 20 nM compared with cyclopamine (IC_50_ = 250 nM) [19]. These observations suggest that SANT75 may be a good candidate as a Hh-targeted antitumor drug. However, SANT75 is water insolubility and acute toxicity, which directly prevented the exploration about the druggability of SANT75. A potential solution is needed to overcome these limitations of SANT75 without decreasing its activity.

Liposomes, artificial phospholipid vesicles with a bilayered membrane structure, which can be loaded with water-soluble drugs into inner aqueous compartment and water-insoluble drugs into hydrophobic compartment, are considered as promising pharmaceutical carriers [20]. During the past few years, liposomes have drawn much attention for their excellent bioavailability, biodegradability, and targeting characteristically to the reticuloendothelial system (RES), especially the liver and spleen, which enhance the therapeutic efficacy and reduce the toxicity of agents [21],[22]. The liposomes are based on a formulation containing egg phosphatidylcholine, cholesterol and dimethyldioctadecyl ammonium bromide and the current liposome research focuses on development of various liposome-based multifunctional nanopreparations for therapy and diagnosis [23],[24]. Polyethylene glycol (PEG) is widely used to modify liposomes, which display inhibited interaction with plasma proteins and mononuclear phagocytes and consequently prolong blood circulation time, decrease nonspecific interaction with the reticuloendothelial system (RES), and improve the size uniformity of particles [25]–[27].

Thus, in the current study, we selected PEGylated liposome as a delivery system to improve *in vitro* and *in vivo* antitumor efficacy of SANT75. We used Shh-light2 cell and transgenic reporter zebrafish to evaluate the activity of SANT75 before and after encapsulating into liposome, and established a liposome-formulated SANT75 that is capable of effective suppressing tumor growth through inhibition of the Hh pathway.

## Materials and Methods

### Ethics statement

All animal work were approved by Sichuan Animal Care and Use Committee and strictly conducted in accordance with relevant guidelines. The Permit Number is SYXK (Chuan) 2008-119.

### Materials

Soybean phosphatidylcholine (SPC), cholesterol (CHOL), and distearoly- phosphatidylethanol-amine-N-poly (ethyleneglycol) 2000(DSPE-PEG 2000) were purchased from Lipoid GmbH Co. (Ludwigshafen, Germany). SANT75 was synthesized as previously described [19]. A rabbit polyclonal antibody against GLI-1 was purchased from Santa Cruz Biotechnology Co. (Santa Cruz, CA). A rat antimouse CD31 monoclonal antibody was purchased from BD Biosciences Co. (PharMingen, San Diego, CA). In situ Cell Death Detection kit (DeadEnd™ Fluorometric TUNEL System) was purchased from Promega Co. (Promega, Madison, WI).

### Cell culture

Tumor cell lines with high-expression of Hh pathway including Murine Lewis lung cancer cell line LL/2, human lung cancer cell lines h460, human ovarian cancer cell line SKOV3, human prostate cancer cell line DU145, human colon cancer cell line SW480 and SW620 were obtained from the American Type Culture Collection (ATCC, Manassas, VA) [6]–[9]. These cells were cultured in DMEM or RPMI-1640 supplemented with 10% fetal bovine serum, 100 units/mL penicillin, and 100 units/mL streptomycin. The Shh-light2 cell reporter system (gift from James Chen, Stanford University) is a NIH-3T3 cell line stably incorporating Gli-dependent firefly luciferase and constitutive Renilla luciferase reporters. These cells were cultured in DMEM containing 10% calf serum, 400 ug/mL geneticin, 200 ug/mL zeocin, 100 U/mL penicillin, and 0.1 mg/mL streptomycin. The Shh-N-producing HEK293 cells, stably transfected with Shh-N expression and neomycin resistance constructs, were cultured in DMEM containing 10% (v/v) FBS and 400 µg/mL G418.All of the cells were maintained in a 37°C incubator with a humidified 5% CO_2_ atmosphere.

### Liposome preparation

Liposomal SANT75 formulations were prepared by the thin-film ultrasonic method. Briefly, the mixtures of SPC/cholesterol/DSPE-PEG2000/SANT75 in 8∶2∶1∶1 weight ratios were dissolved in ethanol and were transferred into a suitable round bottom flask. The flask was then connected to a rotary evaporator at 80 rpm and water bath with temperature maintained at 40°C. Vacuum was applied to the flask to evaporate the ethanol and form a homogeneous lipid film on the flask wall. The trace amount of ethanol was removed under vacuum overnight. The lipid film was then hydrated in normal saline by rotating the flask at 60°C until the lipid film was completely hydrated. The suitable-size liposome was acquired with ultrasound. The preparation of empty liposome was the same as the liposomal SANT75 without SANT75 in the mixtures.

### Liposome characterization

The mean particle size distribution and zeta-potential (ZP) of liposomes were determined using dynamic light scattering on a Malvern ZEN 3600 (Malvern instruments, Malvern, UK) at 25°C after diluted with distilled water with a volume ratio of 1/100. Besides, the polydispersity index (PI) was determined as a measurement of the distribution of nanoparticle population. DTS ver.5.10 software (Malvern Instruments, Malvern, UK) was used to collect the data. The morphology of empty and SANT75 loaded liposome was investigated by a transmission electron microscope (TEM; HITACHI H-600, Japan) in Basic and Forensic Medicine College of Sichuan University.

The HPLC system, consisted of a Waters Alliance 2695 Separations Module, a Waters 2996 Photodiode Array Detector, and a Waters SunFire™ C18 column (4.6×150 mm, 5 µm, Waters Corp., Milford, MA, USA), was used for the analysis of SANT75 and liposomal SANT75 with a mobile phase containing a mixture of 0.1% formic acid and methanol (65∶35, v/v) at a flow rate of 1 ml/min at 25°C column temperature. Sample injection volumes were 10 µl and SANT75 detection was performed using UV detector at 226 nm wavelength.

Entrapment efficiency of SANT75 into liposome was determined by a modified minicolumn centrifugation method using poly-prep chromatography column (Bio-Rad, Hercules,CA, USA) filled with Pharmacia Sephadex G-50 Medium (GE,USA ) to separate free SANT75 from the liposome-entrapped drug as described previously [28]. Briefly, the free liposome was saturated the pre-prepared column to minimize adsorption of actual sample (liposomal SANT75). Then, the liposomal SANT75 sample was introduced into the column and the entrapped liposomal SANT75 was eluted by centrifugation, which was solubilized with 10% Triton X-100(1∶1, v/v) and analyzed for SANT75 concentration using the HPLC system. The entrapment efficiency was calculated by comparing the SANT75 concentration of the eluted sample with that of liposomal SANT75 sample prior to column chromatography. The related equation was:
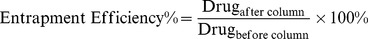



### Liposomal SANT75 activity in Shh-light2 cells

The activity of non-encapsulated and encapsulated SANT75 was assessed by the Shh-light2 cells system for Hh pathway activation. The method was as described previously [29]. The Shh-light2 cells were seeded into 96-well plate using complete medium for 12 h or overnight, and then these cells were treated with various concentrations of liposomal SANT75 or free SANT75 (dissolved in DMSO) in DMEM containing 0.5% CS, 100 U/mL penicillin, 0.1 mg/mL streptomycin, 5% Shh-N-conditioned medium obtained from Shh-N- producing HEK293 cells. After the cells were cultured for an additional 30 h, firefly and Renilla luciferase activities were measured on a Veritas microplate luminometer (Turner Biosystems) using a Dual Luciferase Reporter kit (Promega).

### Liposomal SANT75 activity in zebrafish

Zebrafish were bred and maintained under condition of 28°C; pH 7.2–7.4; 14 hr on and 10 hr off light cycle. The embryos from Gli-GFP or Flk-GFP transgenic zebrafish were distributed to 96-well plates with three embryos placed in each well, and then various concentrations of liposomal SANT75 or free SANT75 were added. The embryos were exposed to drug solution and incubated at 28.5°C from 4 h post-fertilization (hpf) to 48hpf. The phenotypes were observed at 36 and 48hpf using an Axioimager Z1 fluorescence microscope (Zeiss).

### Cell proliferation assay

The growth-inhibitory activity of liposomal SANT75 on LL/2 and other cells was evaluated by 3-(4, 5-dimethylthiazol-2-yl)-2, 5- diphenyltetrazolium bromide colorimetric assay. The LL/2 and other cells cultured in 96-well multi-well plates were exposed to various concentrations of liposomal SANT75 or free SANT75 with equivalent dose of SANT75 for 48 hours. Besides, The LL/2 cell line was treated with free or liposomal SANT75 for various time intervals (24 h, 48 h, 72 h) at equivalent dose of SANT75 (20 µM).The control culture was treated with free liposome without addition of SANT75. Then, 20 µl MTT (5 mg/mL in PBS) was added to each well at different time point, and after incubated for 4 hr, the medium was removed, and 150 µl of dimethyl sulfoxide (DMSO) was added per well. Spectrometric absorbance at 570 nm was measured on Multiscan MK3 ELISA reader (Thermo, USA). The cell survival rate was assessed as percent cell viability in terms of non-treated control cells.

### Pharmacokinetic study

Before administration, SD rats were catheterized with polyethylene tube in the jugular vein under anesthesia with 10% chloral hydrate. The cannula was flushed with heparin sodium to prevent the blood clotting. On the second day, the rats were randomly divided into experimental groups (6 rats per group) for treatment with SANT75 (formulation: formic acid 0.1%, Tween-80 5% in distilled water) or liposomal SANT75 at a dose of SANT75 (5 mg/kg) via jugular vein. After dosing, 0.3 ml blood was collected in heparinized tubes from catheterized jugular vein at 2, 10, 20, 30, 45 min, 1, 2, 4, 6, 8, 10, 12 h, 24 h. The blood samples were then centrifuged at 13000 rpm for 10 min at 4°C to separate plasma and the plasma was kept at −80°C till analysis for SANT75.

The diphenhydramine was added to plasma samples as the internal standard, and acetonitrile as protein precipitator. The high performance liquid chromatography (Waters Quattro Premier XE) with mass spectrometry (Waters Acquity UPLC™) was used for analyzing SANT75 with a Acquity UPLC BEH C18 column (2.1×50 mm, 1.7 µm, Waters Corp., Milford, MA, USA). The mobile phase was a mixture of acetonitrile and 0.1% formic acid (80∶20, v/v), and flow rate of 0.25 ml/min was used. The sample injection volumes were 5 µl. The data was analyzed by the DAS 2.1.1 software.

### Tissue distribution study in tumor-bearing mice

Female C57 mice (n = 40) were inoculated with LL/2 cells, and when tumor volume reached approximately 60 mm^3^ (5×5 mm in diameter), the mice were randomized into 2 groups and treated with free SANT75 or liposomal SANT75 at a dose of SANT75 (20 mg/kg) via caudal vein. After dosing, mice were sacrificed and the tumor, heart, liver, spleen, lung, kidney, intestine were excised at defined time points (5, 60, 120, 240 min). These tissues were then grinded with liquid nitrogen, weighed, and analyzed for SANT75 using the high performance liquid chromatography with mass spectrometry.

### Effect of liposomal SANT75 on Lewis lung carcinoma

Female C57BL/6 mice aged 6–8 weeks were purchased from the Animal Center of Sichuan University and allowed to acclimate for 1 week before use. 1×10^6^ Lewis lung carcinoma cells (LL/2) were inoculated subcutaneously in the right flank of each mouse, and when tumor volume reached approximately 60 mm^3^ (5×5 mm in diameter), the mice were randomized into 3 groups (n = 8 for each group) and treated with liposomal SANT75 (40 mg/kg), free liposome, and normal saline alone, respectively. Each mouse received treatment intravenously injection every 2 day for 15 days (8 injections). And the mice were monitored on a daily basis for tumor burden, general condition, food and water supply.Tumor growth was monitored with calipers every 3 days. Tumor volume was calculated using the formula: volume = 0.52×length×width^2^. These mice were sacrificed 2 days after the last treatment, and tumors were excised, weighed and fixed in 10% neutral buffered formalin solution or frozen at −80°C.

In another group of mice experiment for survival study, the tumor-bearing mice (n = 8 for each group) were medicated as described above, and the mice were sacrificed when they became moribund and the sacrificed date was recorded to calculate the survival time.

### Histological analysis

Paraffin sections from each group were stained with hematoxylin–eosin (H&E). The immunofluorescence of neovascularization was performed as follows: the frozen tissue sections were fixed in acetone, incubated with rat anti-mouse CD31 monoclonal antibody at 4°C overnight and then stained with goat anti-rat IgG/TRITC. The sections were viewed under an Axioimager Z1 fluorescence microscope (Zeiss). Vessel density was determined by counting the number of microvessels per high-power field in sections. Immunostaining for GLI-1 expression in tumor was done as described previously [30]. Briefly, 6 µm frozen sections were fixed in 10% neutral buffered formalin for 30 minutes, and incubated in 3% hydrogen peroxide followed by 1% goat serum at room temperature (RT). Rabbit polyclonal antibody GLI-1 was applied at 4°C overnight and immunoreactivity was visualized using peroxidase-DAB. The hematoxylin was used as a counterstain. The activity of GLI-1 within tumor species was determined by the shade of brown. The deeper the color, the stronger the GLI-1 activity.

### Assessment of apoptosis

The presence of apoptotic cells within tumor species was determined using the In situ Cell Death Detection kit (DeadEnd™ Fluorometric TUNEL System) following the manufacturer's instructions. In paraffin sections, four equal-sized fields were randomly chosen and analyzed. The apoptotic index (AI) was defined as follows: AI (%) = 100×apoptotic cells/total tumor cells.

### Evaluation of potential side effects

In order to investigate the toxicity of liposomal SANT75 compared with SANT75 (formulation: DMSO: Tween-80: Saline = 10∶5∶85, v/v/v), C57BL/6 mice were treated i.v. with liposomal SANT75 (20 mg/kg, 40 mg/kg), SANT75 (20 mg/kg, 40 mg/kg), solvent, and saline respectively every day till appearance of swollen tails. The severity of tail swelling in different groups was analyzed with tail circumference.

The weight loss, life span, and behavior were investigated in LL/2-bearing C57BL/6N mice treated with liposomal SANT75. Tissues of heart, liver, spleen, lung, and kidney were also fixed in 10% neutral buffered formalin solution for a pathological examination.

### Statistical analysis

Data was assayed by ANOVA and unpaired student's t-test. Differences between means or ranks as appropriate were considered significant when p value is <0.05. The SPSS statistics 17.0 and GraphPad Prism 5 were used for statistical analyses.

## Results

### Preparation and characterization of liposomal SANT75

**Figure 1:**
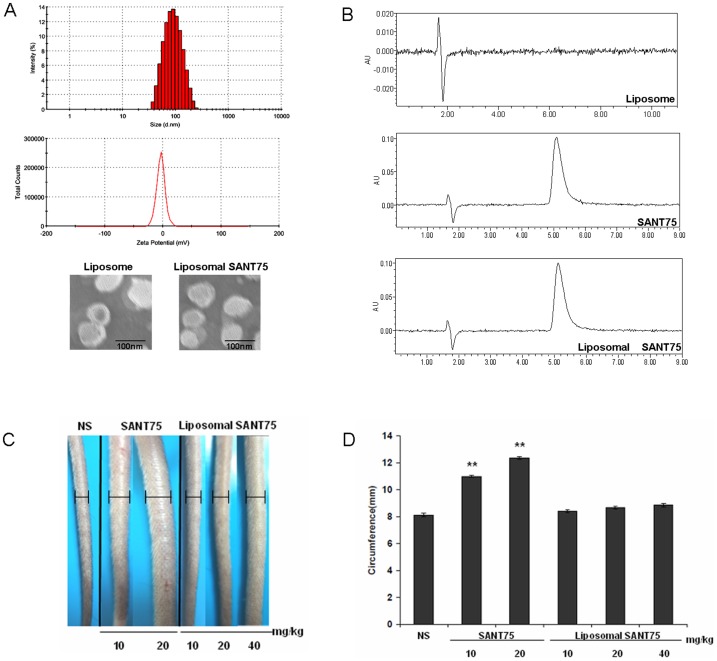
The characterization and the decreased toxicity of liposomal SANT75. (A) Size distribution, zeta potential spectrum and the morphologic photomicrographs of liposomal SANT75. (B) The HPLC analysis of free liposome, SANT75, and liposomal SANT75. (C) Photograph of mice tails after i.v. of free SANT75 and liposomal SANT75 with various doses. (D) The statistics of the mouse tail circumferences reflecting the severity of swelling in different groups. Columns, mean; bars, SD. **, P<0.01, SANT75 group versus the normal saline (NS) or liposomal SANT75 group.

Liposomal SANT75 synthesized by the thin-film ultrasonic method was shown to be water-soluble. The physical characteristics of liposome such as particle size, polydispersity index (PI) and zeta potential was investigated before and after encapsulation of SANT75 (Figure 1A). There was no significant difference in mean particle sizes between the free liposome and liposomal SANT75, both with a size about 100 nm. The PI of liposomes was less than 0.2, which showed that liposomes had homogenous size distribution regardless of entrapment of SANT75. The zeta potential of liposomal SANT75 was −3.49±7.34 mV. Transmission electron microscopy examination confirmed that the particle size of empty and drug loaded liposomes was approximately 100 nm, and the images also showed that the liposomes were dispersed and spherical (Figure 1A). The percentage of the drug entrapped into liposomes was estimated to be 87% by the minicolumn centrifugation method. There was no change of the prosperity of SANT75 after encapsulated into liposomes by the HPLC analysis and the retention time was 5.3 min (Figure 1B).

### Decreased toxicity of liposomal SANT75

In our earlier preliminary examination exploring antitumor potent of SANT75, we found that SANT75 had great inhibitory potency in several tumor cell lines, and to further validate its antitumor efficacy *in vivo*, we injected SANT75 (formulation: DMSO: Tween-80: Saline = 10∶5∶85, v/v/v) into tumor-bearing mice via caudal vein at a dose of SANT75 (10 mg/kg, 20 mg/kg, or 40 mg/kg). But severe swelling appeared, and some mice even died after three or four days' treatment, which directly prevented further exploration about the druggability of SANT75 in the mouse model. To assess whether SANT75 encapsulated into liposomes reduce the acute toxicity and allow i.v. administration, we compared the severity of tail swelling after i.v. liposomal SANT75 or free SANT75. There was no obvious swelling appearance after i.v. liposomal SANT75 while the tails swelled extensively after i.v. free SANT75 (Figure 1C). The severity of tail swelling in different groups was analyzed by tail circumference (calculated by diameter) (Figure 1D).The mice also started to die when the dose of free SANT75 was up to 40 mg/kg. This suggested that liposomal SANT75 could be administered by i.v. injection without acute toxicity.

### Characterization of liposomal SANT75 in mammalian cells

**Figure 2.Characterization:**
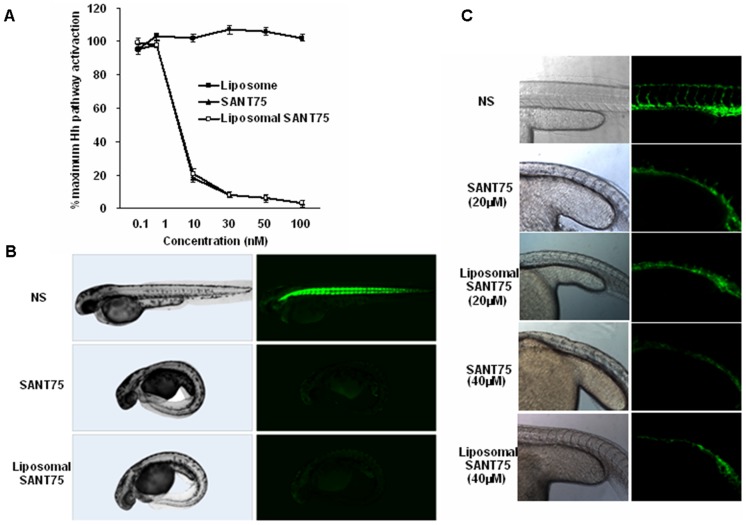
of liposomal SANT75 in Shh-light2 cell and transgenic zebrafish assays. (A) Inhibition of Hh activation by liposomal SANT75 and free SANT75 in SHh-light2 cells. IC_50s_ for both are approximately 5 nM. (B) Inhibition of Hh activity *in vivo* by liposomal SANT75 and free SANT75 using Gli-GFP reporter in transgenic zebrafish embryos. (C) Inhibition of inter-segmental blood vessel by liposomal SANT75 and free SANT75 in Flk-GFP transgenic zebrafish embryos.

To investigate whether liposomal SANT75 keep the same inhibitory potency in Hh pathway as that of free SANT75, Shh-light2 cells cultured in Shh-N-conditioned medium were treated with various concentrations of liposomal SANT75 or free SANT75 with equivalent doses, and the luciferase reporter activities were measured. The results showed that the inhibitory potency of SANT75 remained unchanged after encapsulated into liposome (Figure 2A).

### Characterization of liposomal SANT75 in zebrafish

To assess whether SANT75 encapsulated into liposome keep the similar inhibition of Hh pathway *in vivo*, Gli-GFP and Flk-GFP transgenic zebrafish were used. The Gli-GFP expression in zebrafish was a reflection of the endogenous activity of the Hh pathway [19]. We observed the same GFP expression inhibition in the transgenic zebrafish after treating with liposomal SANT75 or free SANT75, both effective at 5 µM (Figure 2B). The characteristic phenotypes of zebrafish embryos that were deficient of Hh signaling include U-shaped somites and shorter intersegmental blood vessels (ISV) [19]. Tg (Flk1: EGFP) transgenic zebrafish that have vascular endothelial cells labeled by Flk-GFP were treated with liposomal SANT75 or free SANT75. In this assay, action of liposomal SANT75 is dose-dependent, which is similar to free SANT75. The embryos treated with 20 µM liposomal SANT75 from 2hpf to 36hpf produced U-shaped somites and reduced the sprouting of ISV. When the dose of liposomal SANT75 was up to 40 µM or above, the embryos displayed strong developmental deficiencies (Figure 2C). These data further validated that SANT75 inhibited Hh pathway whether encapsulated into liposome or not.

### Pharmacokinetics and tissue distribution of liposomal SANT75

**Figure 3:**
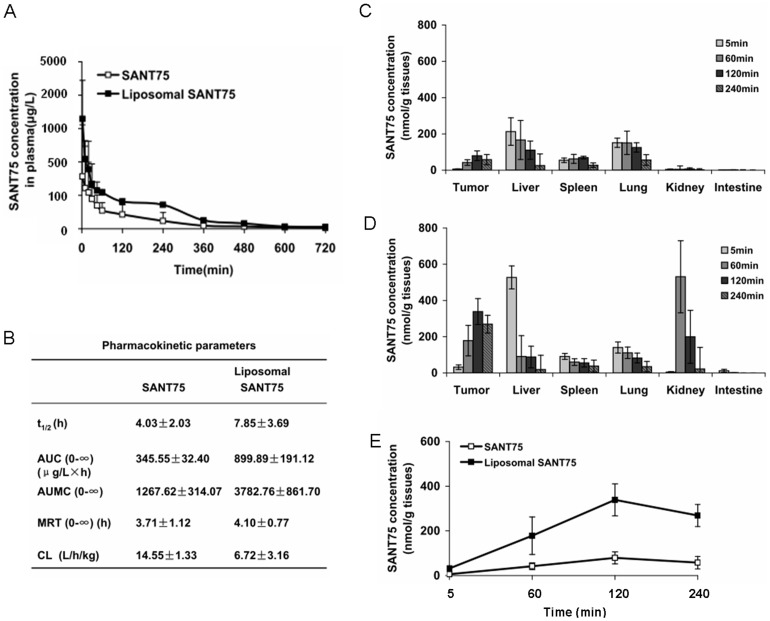
Pharmacokinetics and tissue distribution of liposomal SANT75. (A) The concentration - time curve of SANT75 in plasma after intravenous administration of liposomal SANT75 or free SANT75 to rats. (B) Pharmacokinetic parameters after intravenous administration of liposomal SANT75 or free SANT75 to rats. The results are expressed as the mean ± SD. (C) The tissue distribution of free SANT75 in tumor-bearing mice at different time point. Columns, mean; bars, SD. (D) The tissue distribution of liposomal SANT75 in tumor-bearing mice at different time point. Columns, mean; bars, SD. (E) The drug distribution of free SANT75 or liposomal SANT75 in tumor tissues at different time point.

To investigate whether the liposomal formulation improve bioavailability of SANT75, after dosing liposomal SANT75 or free SANT75, the blood samples and tissues as well as tumors were collected and the SANT75 concentration at various times was analyzed using HPLC-MS. The SANT75 concentration in plasma-time profiles of liposomal SANT75 and free SANT75 injection were illustrated (Figure 3A), and all the plasma concentration-time data were fitted with a three-compartment model. The liposomal SANT75 increased the pharmacokinetic parameters of SANT75 such as AUC (0-∞), AUMC (0-∞) and half-life, but reduced CL compared to SANT75 (Figure 3B). The increased AUC confirmed slower SANT75 removal from the plasma compartment of SANT75-encapsulated liposomes. The incorporation of SANT75 into liposomes prolonged biological half-life of SANT75. The CL of liposomal SANT75 was 6.72 L/h/kg while free SANT75 was 14.55 L/h/kg. When encapsulated into liposome, SANT75 was highly distributed in tissues especially in tumors compared with the mice treated by free SANT75, which indicated that the liposome formulation improved SANT75 tumor-targeted effect (Figure 3C–E).These results suggested that the bioavailability and antitumor efficacy of SANT75 has been improved after encapsulated into liposomes.

### Inhibition of tumor cell proliferation

**Figure 4:**
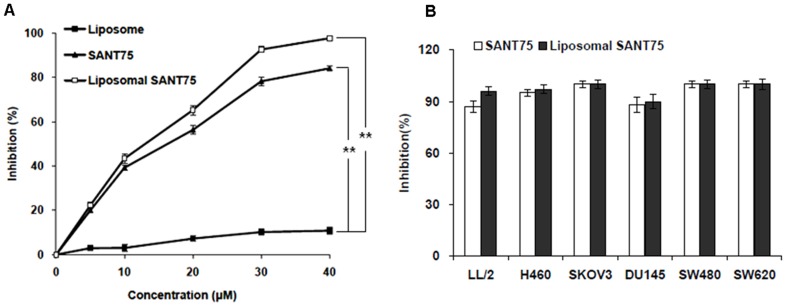
Inhibition of tumor cell proliferation treated with liposomal SANT75, free SANT75 or free liposome. (A) Concentration-dependent inhibition of proliferation of LL/2 cells by different formulations. **, P<0.01, liposome group versus free SANT75 or liposomal SANT75 group. (B) The inhibition rate of LL/2, H460, Skov3, DU145, SW480, and SW620 tumor cell lines by liposomal SANT75 or free SANT75 at equivalent dose of 40 µM.

As the Hh pathway inhibitors have shown great promise in cancer therapeutics, we hypothesized that liposomal SANT75 could be a potent antitumor candidate. We first assessed the inhibitory effect of liposomal SANT75 in tumor cells. Lewis lung carcinoma cell and other cell lines that have been reported to have high Hh expression (6–9) were treated with various doses of liposomal SANT75. The liposomal SANT75 treatment resulted in similar antiproliferative effect compared with free SANT75. The inhibition effects were dependent on the dose of SANT75. When LL/2 cells were treated for 48 hours, the inhibition ratio of 5 µM and 20 µM liposomal SANT75 was 22% and 65.3%, respectively (Figure 4A). Similar results were found in LL/2 and other cell lines when treated them with the same dose of SANT75 (40 µM) in different formulations (Figure 4B).And there was no obvious time-dependent inhibition effect existed in LL/2 cell line when treated by liposomal SANT75 for various time intervals, which was consistent with free SANT75 (Figure S1). These data indicated that liposomal SANT75 inhibited tumor proliferation *in vitro* and the inhibitory efficacy was similar to free SANT75.

### *In vivo* antitumor activity

**Figure 5:**
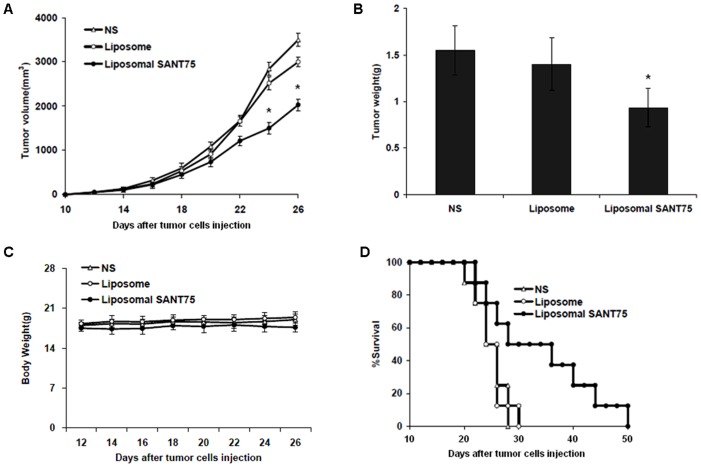
Intravenous administration of liposomal SANT75 inhibited the growth of Lewis lung carcinoma *in vivo* and prolonged the survival of treated mice. (A) Suppression of tumor growth measured by volume in each treatment group. Points, mean; bars, SD. *, P<0.05, liposomal SANT75 group versus the normal saline or liposome group. (B) Tumor weight of subcutaneously transplanted tumors in each treatment group. Columns, mean; bars, SD. *, P<0.05, liposomal SANT75 group versus the normal saline or liposome group. (C) The body weight curves of mice in each group. (D) Survival curves of mice in each group.

To further validate antitumor efficacy of liposomal SANT75 *in vivo*, we next established the homograft tumor model. C57BL/6N mice bearing LL/2 lewis lung carcinoma received i.v. administration of liposomal SANT75 every other day for 15 days. Treatment with liposomal SANT75 resulted in effective suppression of tumor growth versus controls (*P*<0.05, Figure 5A), and the mean tumor weight in liposomal SANT75 group was 0.9 g compared with that in empty liposome group, which had a mean weight of 1.4 g (Figure 5B). No obviously adverse effects were observed throughout the whole experiment and mice showed no significant change in body weights (Figure 5C). In addition, survival time was greatly prolonged in mice that received liposomal SANT75 (mean = 50 days) compared with the normal saline (mean = 29 days) or liposome (mean = 30 days) (Figure 5D). These data determined that the antitumor activity of liposomal SANT75 *in vivo* was not attributable to systemic toxicity.

### Liposomal SANT75 induced apoptosis

**Figure 6:**
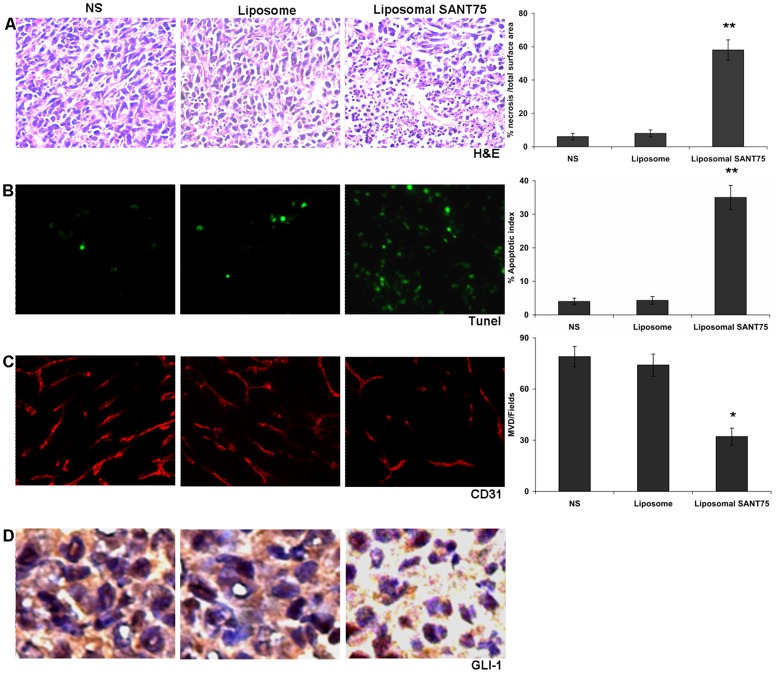
Histochemical analysis of tumor tissues in the normal saline group, free liposome group and liposomal SANT75 group. (A) H&E staining of the tumor tissues in each treatment group. Data represent the mean necrosis index ± SDs of cancer cells. Columns, mean; bars, SD. **, P<0.01, significantly different from the normal saline or liposome group. (B) TUNEL staining of the tumor tissues in each treatment group. Data represent the mean apoptotic index ± SDs of cancer cells. Columns, mean; bars, SD. **, P<0.01 significantly different from the normal saline or liposome group. (C) Inhibition of angiogenesis assayed by immunofluorescence staining with CD31. The number of vessels was counted as described in the methods. Columns, mean; bars, SD. *, P<0.05 liposomal SANT75 group versus the normal saline or liposome group. (D) The Gli-1 expression by immunohistochemical detection in each treated tumor tissues.

The H&E staining was conducted in order to investigate the pathological morphology in tumor tissues. Compared with controls, tumors treated with liposomal SANT75 showed more extensive necrosis areas, which was characterized by the presence of fragmented nuclear and cytoplasmic debris and a nearly complete lack of intact cells [31] (Figure 6A). To address if the cell death resulted from apoptosis, tumor tissues were subjected to TUNEL assays, which revealed higher apoptosis rate induced by liposomal SANT75 compared with controls (*P*<0.05) (Figure 6B).

### Liposomal SANT75 inhibited tumor vascularization

The previous study indicated that inhibiting Hh signaling decreased tumor angiogenesis [32].To assess the effect of liposomal SANT75 on microvessel density (MVD) in the grafted tumors, immunofluorescence staining for CD31 was performed. The most highly vascularized area of each tumor was identified under a low power (5×) microscope objective, and five high-power (40×) fields were counted in this area to quantify vessel density. This assay revealed that liposomal SANT75 therapy significantly reduced the number of tumor microvessels compared with control groups treated with saline and empty liposome (*P*<0.05) (Figure 6C).

### Liposomal SANT75 decreased Gli-1 expression

To further validate liposomal SANT75 inhibited the Hh pathway in tumor tissues, expression of the target gene Gli-1 in the Hh pathway was detected. The immunostaing using an antibody against Gli revealed that liposomal SANT75 decreased the expression of Gli-1 in comparison with controls (Figure 6D).

## Discussion

SANT75 is a Hh pathway antagonist that specially inhibits SMO protein through inducing its conformational change [19]. Although SANT75's anti-Hh activity is highly potent and specific, it has some unfavorable characteristics in pharmacokinetics, limiting its potential as a therapeutic agent. Here, we explored the liposomal formulation of SANT75 to improve its druggability.

Over the past few decades, liposomes have been investigated for the systemic delivery of anti-cancer agents as potential drug delivery systems (DDS) and several drugs with liposomal delivery systems have been approved for clinic applications. For example, liposome-encapsulated doxorubicin (Doxil ®) produced less cardiotoxicity than free doxorubicin while providing comparable antitumor activity [33],[34]. In the current study, we used PEGylated liposome as the delivery system of SANT75. We also added distearoly- phosphatidylethanol- amine -N -poly (ethyleneglycol) -2000 (DSPE-PEG 2000) into the liposome formulation to prepare a novel PEGylated liposome-based SANT75. The prepared liposomal SANT75 sized about 100 nm with a PI value less than 0.2 and the zeta potential of liposomal SANT75 was −3.49±7.34 mV. Liposomes of about 100 nm in diameter have been shown to be optimal for the delivery of anticancer drugs to tumors, and a small value of PI (<0.2) indicates a homogenous vesicle population [35],[36].

The PEGylated liposomes have several advantages for pharmaceutical applications, i.e. high water solubility, lack of toxicity and immunogenicity and rapid clearance from the body [36]. Liposomal SANT75 dispersed in water easily and allowed i.v. administration without causing swelling. Previous studies identified that anticancer agents encapsulated by PEGylated liposome can reduce the uptake by macrophages and RES because of steric stabilizing effect of PEG on liposome surface and significantly prolong the plasma residence time of drugs [37],[38].The concentration in plasma-time profiles and the tissue distribution suggested that the bioavailability and antitumor efficacy of SANT75 have been improved after encapsulated into liposomes.

In our previous study, we have developed an effective phenotype-based transgenic zebrafish embryo assay coupled with mammalian cell assay for identifying and characterizing Hh signaling pathway inhibitors. In the current study, we used the same Shh-light2 cell assay and established that liposomal SANT75 had similar inhibitory potency as that of free SANT75. Also, liposomal SANT75 inhibited Gli-GFP expression at 5 µM and reduced the sprouting of ISV at 20 µM, which were consistent with the previous observations for free SANT75 analyzed by zebrafish assays [19].

The results of our experiments demonstrated that SANT75 encapsulated into liposome exerted strong tumor growth-inhibiting effects *in vitro* and *in vivo*. In addition, the liposomal SANT75 therapy efficiently improved the survival time of tumor-bearing mice without obvious systemic toxicity. The mechanisms of action of liposome-SANT75 appear multifaceted. Firstly, targeting Hh (as shown by reduction of Gli protein) could directly induce apoptosis of tumor cells. Secondly, when Hh pathway was inhibited, tumor angiogenesis were reduced, blocking the nutrition supply into tumor tissue, which promoted apoptosis of tumor cells.

## Conclusion

We prepared a novel antitumor agent using PEGylated liposome as the delivery system to encapsulate SANT75. The liposomal SANT75 could be directly dispersed, allowing i.v. administration without detectable side effects. The liposome-SANT75 maintained the same inhibitory activity of Hh as the free SANT75 in both Shh-light2 cell and transgenic zebrafish assays. The SANT75 encapsulated into liposome is effective in inhibiting tumor cell growth *in vitro* and *in vivo* and may be considered as a new Hh-targeted cancer therapy agent.

## Supporting Information

Figure S1 **Inhibition effect of LL/2 treated with liposomal SANT75, free SANT75 or free liposome at a dose of SANT75 (20 µM) for various time intervals.**
(TIF)
