# Supplementary figures and images for: A Novel PEGylated Liposome-Encapsulated SANT75 Suppresses Tumor Growth through Inhibiting Hedgehog Signaling Pathway
Source: PLoS One. 2013 Apr 1;8(4):e60266. doi: 10.1371/journal.pone.0060266 (PMC3613365; doi:10.1371/journal.pone.0060266)

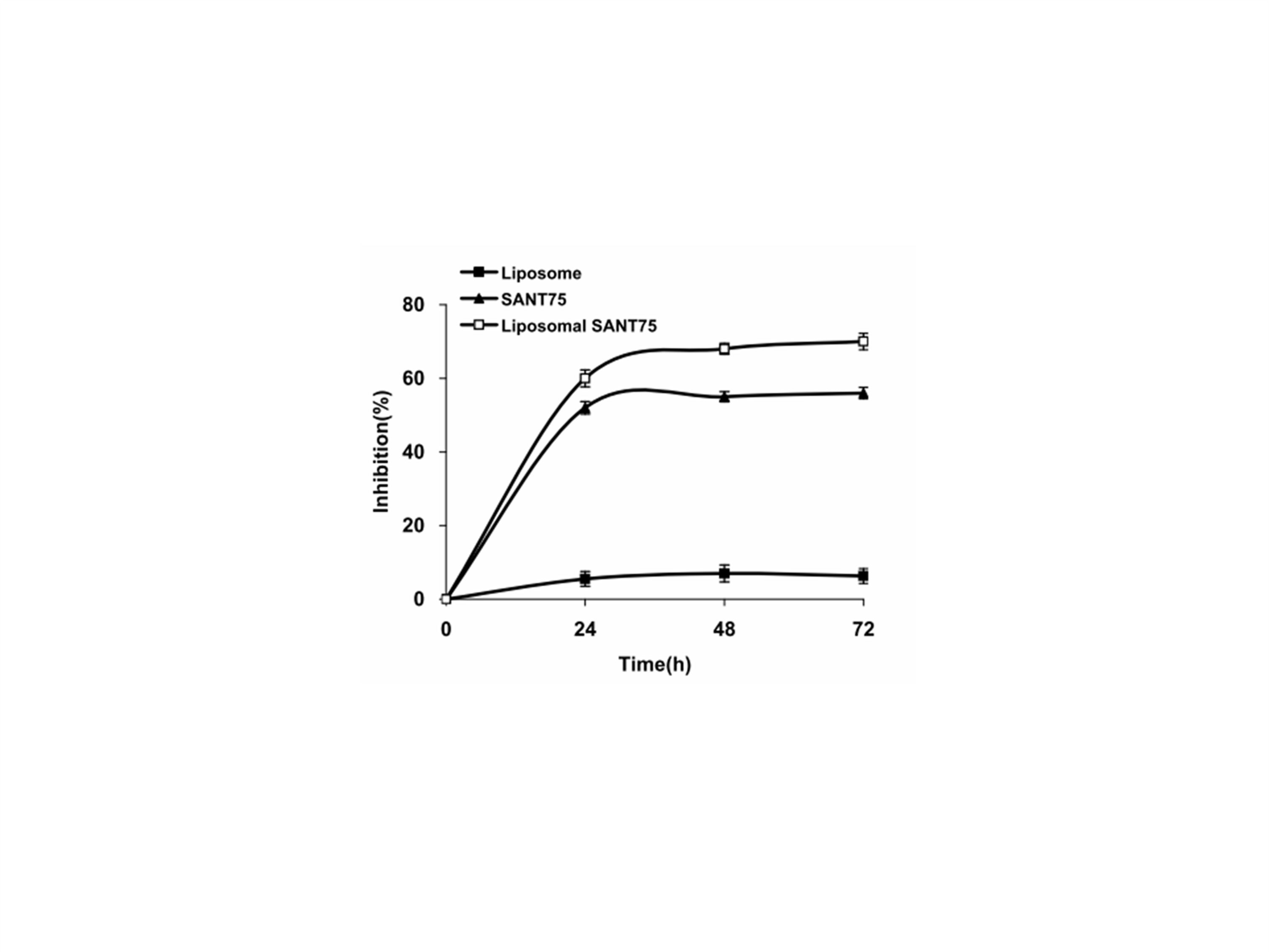

Supplement: Figure S1 — Inhibition effect of LL/2 treated with liposomal SANT75, free SANT75 or free liposome at a dose of SANT75 (20 µM) for various time intervals. (TIF) [file pone.0060266.s001.tif]
